# Sharing the slope: depth partitioning of agariciid corals and associated *Symbiodinium* across shallow and mesophotic habitats (2-60 m) on a Caribbean reef

**DOI:** 10.1186/1471-2148-13-205

**Published:** 2013-09-23

**Authors:** Pim Bongaerts, Pedro R Frade, Julie J Ogier, Kyra B Hay, Judith van Bleijswijk, Norbert Englebert, Mark JA Vermeij, Rolf PM Bak, Petra M Visser, Ove Hoegh-Guldberg

**Affiliations:** 1 School of Biological Sciences, The University of Queensland, 4072 St Lucia, QLD, Australia; 2 Global Change Institute, The University of Queensland, 4072 St Lucia, QLD, Australia; 3 ARC Centre of Excellence for Coral Reef Studies, The University of Queensland, 4072 St Lucia, QLD, Australia; 4 CARMABI, Piscaderabaai z/n, PO Box 2090, Willemstad, Curaçao; 5 Department of Marine Biology, University of Vienna, Althanstrasse 14, 1090 Vienna, Austria; 6 Netherlands Institute for Sea Research, PO Box 59, 1790 Den Burg, AB, The Netherlands; 7 Heron Island Research Station, The University of Queensland, 4680 Heron Island, QLD, Australia; 8 Aquatic Microbiology, Institute for Biodiversity and Ecosystem Dynamics, University of Amsterdam, PO Box 94248, 1090 Amsterdam, GE, The Netherlands

**Keywords:** Niche partitioning, Species diversification, Mesophotic, Deep reef, Agariciidae, *Symbiodinium*

## Abstract

**Background:**

Scleractinian corals and their algal endosymbionts (genus *Symbiodinium*) exhibit distinct bathymetric distributions on coral reefs. Yet, few studies have assessed the evolutionary context of these ecological distributions by exploring the genetic diversity of closely related coral species and their associated *Symbiodinium* over large depth ranges. Here we assess the distribution and genetic diversity of five agariciid coral species (*Agaricia humilis*, *A*. *agaricites*, *A*. *lamarcki*, *A*. *grahamae*, and *Helioseris cucullata*) and their algal endosymbionts (*Symbiodinium*) across a large depth gradient (2-60 m) covering shallow to mesophotic depths on a Caribbean reef.

**Results:**

The five agariciid species exhibited distinct depth distributions, and dominant *Symbiodinium* associations were found to be species-specific, with each of the agariciid species harbouring a distinct ITS2-DGGE profile (except for a shared profile between *A*. *lamarcki* and *A*. *grahamae*). Only *A*. *lamarcki* harboured different *Symbiodinium* types across its depth distribution (i.e. exhibited symbiont zonation). Phylogenetic analysis (*atp6*) of the coral hosts demonstrated a division of the *Agaricia* genus into two major lineages that correspond to their bathymetric distribution (“shallow”: *A*. *humilis* / *A*. *agaricites* and “deep”: *A*. *lamarcki* / *A*. *grahamae*), highlighting the role of depth-related factors in the diversification of these congeneric agariciid species. The divergence between “shallow” and “deep” host species was reflected in the relatedness of the associated *Symbiodinium* (with *A*. *lamarcki* and *A*. *grahamae* sharing an identical *Symbiodinium* profile, and *A*. *humilis* and *A*. *agaricites* harbouring a related ITS2 sequence in their *Symbiodinium* profiles), corroborating the notion that brooding corals and their *Symbiodinium* are engaged in coevolutionary processes.

**Conclusions:**

Our findings support the hypothesis that the depth-related environmental gradient on reefs has played an important role in the diversification of the genus *Agaricia* and their associated *Symbiodinium*, resulting in a genetic segregation between coral host-symbiont communities at shallow and mesophotic depths.

## Background

Historically, coral species were thought to be extremely ecologically and morphologically plastic, because of the broad variation observed “within species” [1]. Advances in coral taxonomy and ecology have demonstrated that this variation often reflects sibling species occupying distinct ecological niches [2]. The recent advent of molecular approaches has exposed a further wealth of cryptic diversity in scleractinian corals [3-5] often in relation to particular environments, highlighting the role of niche partitioning [6-9] in addition to ecophenotypic plasticity [10,11]. Likewise, the algal endosymbionts that associate with reef-building corals and allow them to exploit light as an energy source in oligotrophic conditions, were originally thought to consist of a single algal symbiont, *Symbiodinium microadriaticum*[12]. By now, we know that *Symbiodinium* diversity in tropical scleractinian corals consists of several different phylogenetic clades [13-15], species [16,17] and hundreds of different subclades based on the ITS2 region of the ribosomal DNA [14,16]. These different *Symbiodinium* genotypes often represent physiologically distinct entities that can be strongly partitioned across host species, geographic regions and particular reef environments [18-23]. Although ecological niche partitioning across environmental gradients (e.g. light, temperature, turbidity) appears fundamental to maintaining the diversity of both scleractinian corals and their endosymbiotic dinoflagellates, the evolutionary context of this host-symbiont niche partitioning remains poorly understood.

Despite the great diversity of *Symbiodinium* types found in reef invertebrates, many coral host species appear to associate with a specific genetic lineage (or group) of *Symbiodinium*[16]. Particularly in brooding corals, where the onset of symbiosis occurs through the vertical transmission of *Symbiodinium* from the maternal colony to the larvae, high levels of host-symbiont specificity have been observed [8,24-27]. Molecular studies targeting both symbiotic partners have identified tightly coupled host-symbiont associations in brooding corals at the family [20], genus [9] and species level [8,9,26]. As such, natural selection may be acting on the level of the coral holobiont (i.e., host and symbiont combined) in species with a vertical symbiont acquisition mode, resulting in coevolution of both symbiotic partners. Incongruence between the phylogenies of corals and their associated *Symbiodinium* indicates that recombination of host-symbiont associations does occur over the course of evolution [13,16,28,29], however the occurrences of such events may be less likely over ecological timescales. It is therefore important to study the evolutionary origin and implications of such host-symbiont specificity, particularly in light of the environmental change predicted over the next decades [30].

The evolutionary context of coral host-symbiont niche partitioning is also important in the context of reef connectivity between distinct environments and more specifically the ability of reef habitats that offer some kind of protection against certain disturbances, such as deep reefs, to act as a “protected” local source of propagules [31,32]. Although the association of a coral species with different *Symbiodinium* types may facilitate its distribution across distinct reef environments [20,33-35], larval connectivity between these alternative environments may be hampered if symbiotic associations are constrained through vertical symbiont transmission and recruits are not able to adopt symbiont types better-suited to their “new” environment [26]. Along those same lines, host populations may be locally adapted to opposing environments and distinct symbiont populations may in fact reflect genetic differentiation of both symbiotic partners [31]. Such genetic partitioning of host and symbiont populations has been observed across shallow and deep populations of the pocilloporid coral species *Madracis pharensis* in the Caribbean [9] and *Seriatopora hystrix* on the Great Barrier Reef [8,26,32]. To gain a better understanding of the ability of mesophotic reefs to act as refugia (for species that occur over large depth ranges) and a subsequent source of reproduction for degraded shallow reefs it is important to determine the overlap in genetic diversity of host and symbiont communities in shallow and deep reef environments. Given the prevalence of a brooding reproductive mode (>65%) and therefore vertical symbiont transmission on Caribbean reefs [31,36], there is a potential for strong genetic differentiation between host-symbiont associations in shallow and mesophotic communities in this region [27]. However, this hypothesis remains largely untested, primarily as molecular data from mesophotic coral communities are limited.

Mesophotic reef communities in the Caribbean are commonly dominated by members of the Agariciidae [37-39], and in particular by the species *Agaricia lamarcki* and *Agaricia grahamae*[31,40]. The plating growth forms, such as those of Agariciidae, are thought to be particularly well suited to maximize light capture in these low-light environments [41]. Nonetheless, species of the genus *Agaricia* can be abundant throughout the entire depth range of Caribbean reefs and *A*. *humilis*, for example, occurs only in very shallow sections of reefs [42]. Species of *Agaricia* can exhibit high linear growth rates [43] and these brooding species are major contributors to the pool of coral recruits on Caribbean reefs [38,42,44,45]. Data on the phylogenetic affiliation of *Symbiodinium* associated with Western-Atlantic agariciids is limited, however records so far demonstrate association with *Symbiodinium* from clade C and occasionally clade D [16,46-50]. It remains unclear whether the brooding reproductive mode of Western-Atlantic agariciids has resulted in host-symbiont specificity and to what extent the associated *Symbiodinium* diversity allows this genus to be abundant across large depth gradients.

Here, we assessed *Symbiodinium* genetic diversity for the five most common agariciid corals in Curaçao over a large depth range (2-60 m) encompassing both shallow and mesophotic depths. We addressed the level of specificity between the different coral species and their dominant *Symbiodinium* (i.e. the algal type(s) that are most abundantly present in the coral tissue), and evaluated whether these host-symbiont associations are partitioned over depth. Additionally, sequencing of a host mitochondrial region disclosed agariciid evolutionary patterns, and allowed evaluation of further genetic structuring of the coral host in relation to symbiont and depth. Results are discussed in light of extensive previous work on the brooding species of the coral genus *Madracis*[9,21,34,51] to address common patterns of genetic diversity over depth, and explore potential processes of codiversification in brooding Caribbean corals and their algal symbionts. Overall, the potential role of depth-related processes in the evolution of brooding corals and their associated *Symbiodinium* is discussed, as are the implications with regards to larval connectivity between shallow and deep coral reef habitats.

## Methods

### Study site and environmental conditions

**Figure 1:**
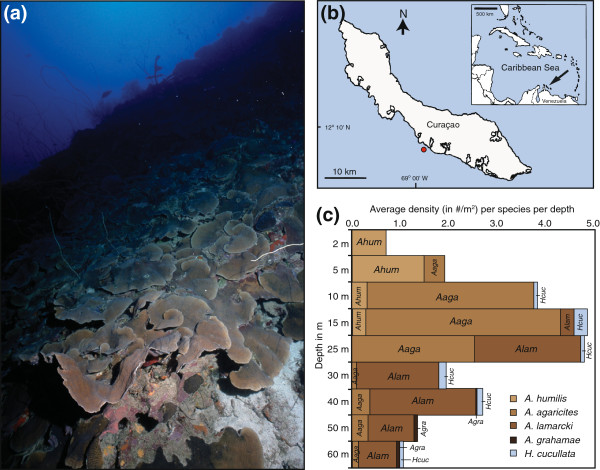
**Mesophotic habitat, sampling location, and agariciid abundances over depth. (a)** Mesophotic *Agaricia* communities on the leeward shore of Curaçao at 50 m depth, **(b)** location of Curaçao (indicated by arrow) and study site Buoy Zero/One (indicated by red dot), and **(c)** distribution of *Agaricia* spp. and *Helioseris cucullata* over depth at the Buoy Zero/One study site.

Collections were performed between 2008 and 2009 at the well-studied site Buoy Zero/One (Bak 1977; Bak et al. 2005; Vermeij et al. 2007; Frade et al. 2008a) in Curaçao, southern Caribbean (12°07’31” N, 68°58’27”W) (Figure 1a). Information obtained from additional samples of previous collections at the same study site in 2004 and 2007 were added to the dataset. The study site is, as are many leeward sites on Curaçao, characterized by a shallow terrace (50-100 m wide) that slopes down to 8-12 m, followed by a steep drop-off with a slope extending down to a deep terrace at 50-60 m depth [52]. Environmental conditions, including seawater temperature and light attenuation were characterized during the beginning of the sampling period and described in Frade et al. [9]. Downwelling light availability (or PAR irradiance) decreases exponentially with a K_d_s of 0.07 m^-1^[9], leading to ~87% of surface irradiance at 2 m, 70% at 5 m, 50% at 10 m, 17% at 25 m, 6% at 40 m, 3% at 50 m, and 1.5% at 60 m. Temperature at the study site shows seasonal variation, with mean seawater temperatures decreasing over depth (0.02°C per metre) [9]. The deeper reef slope (40-60 m) experiences cold-water influxes due to thermocline movements, which can cause a sudden decrease in temperature of several degrees [9,39].

### Agariciid species

The five most common agariciid species in Curaçao, *Agaricia humilis* Verrill, 1901, *A*. *agaricites* (Linnaeus, 1758), *A*. *lamarcki* Milne Edwards and Haime, 1851, *A*. *grahamae* Wells, 1973, and *Helioseris cucullata* (Ellis and Solander, 1786) were included in this study. Of the other three Caribbean agariciid species, *A*. *undata* (Ellis and Solander, 1786) is rare, and *A*. *fragilis* Dana, 1848 and *A*. *tenuifola* Dana, 1848 are absent on the reefs of Curaçao. Coral species were identified following the taxonomic features specified by Wells [53], Veron and Stafford-Smith [54] and Humann & Deloach [55] (Table 1). Fragments of colonies that were collected but that did not clearly conform to these species descriptions were labeled as “*Agaricia* sp.”.

**Table 1:** Morphological characteristics of the five studied Agariciid species

**Species**	**n**	**Colony shape**	**Plates**	**Corallites**	**Valley orientation**	**Septocostae**	**Other**
*Agaricia humilis*	71	Submassive, encrusing, or plating	n.a.	Deep, monocentric corallites (or 2-3 centres together)	Centres separated by collines of irregular height running in irregular directions	Alternating	
*Agaricia agaricites*	113	Encrusting, or plating	Bifacial (fronds)	Short, deep valleys (usually <50 mm).	Irregular, or concentric on plates		
*Agaricia lamarcki*	105	Plating	Unifacial	Valleys with widely spaced centres	Concentric valleys	Alternating	White & star-shaped mouths
*Agaricia grahamae*	20	Plating	Unifacial	Long, V-shaped valleys	Roughly concentric valleys	Non-alternating	
*Helioseris cucullata*	16	Plating	Unifacial	Non-continuous valleys with centres nested against steep ridges	Roughly concentric valleys	Alternating	

Taxonomic features of the different Caribbean Agariciid species studied here, as specified by Wells [53], Veron and Stafford-Smith [54] and Humann & Deloach [55].

### Species abundances and sampling approach

Species abundances of the different agariciid species were determined in 2009 by counting all individuals in 30-50 quadrats (1 × 1 m) laid out randomly (i.e. using a list of randomly generated numbers) along a 100 m long transect parallel to the shore at each sample-depth (2, 5, 10, 25, 40, 50, and 60 m). A total of 335 agariciid coral colonies were sampled over a depth range from 2 to 60 m from a single reef location (Buoy Zero/One), by removing a small fragment from the colony (~3 cm^2^). Fragments were collected at depth intervals that reflect the bathymetric distributions of each species: *A*. *humilis* at 2, 5 and 10 m (n = 71); *A*. *agaricites* at 5, 10, 15, 25, 30, 40 and 50 m (n = 113); *A*. *lamarcki* at 10, 25, 40 and 50 m (n = 105); *A*. *grahamae* at 50 and 60 m (n = 20); and *H*. *cucullata* at 25 and 40 m (n = 16). Tissue from collected coral samples was separated from the skeleton using a modified airgun attached to a SCUBA cylinder or with a sterilized scalpel, and was subsequently stored in 20% DMSO/EDTA preservation buffer or 95% ethanol at -20°C until further processing. DNA was extracted from the tissue using a Qiagen Plant Mini Kit, MoBio Ultra Clean Soil DNA Kit (following the manufacturer’s instructions), or a slightly modified method used for black tiger shrimp [32,56].

### *Symbiodinium* ITS2 analyses

The internal transcribed spacer (ITS2) region of *Symbiodinium* rDNA was amplified for all specimens (n = 335) using *Symbiodinium*-specific primers [46] as described in Bongaerts et al. [8]. The amplified ITS2 fragments were separated using denaturing gradient gel electrophoresis (DGGE) on a CBScientific System following conditions outlined in Sampayo et al. [20], to identify the dominant *Symbiodinium* types in each sample (ITS2-DGGE fails to detect symbionts present in low abundances [57,58]). On each DGGE gel, we ran representative samples to allow for profile cross-comparison and identification. Representative, dominant bands of each characteristic profile were excised (from several replicate profiles), eluted overnight in dH_2_O, re-amplified and purified (using ExoSAP-IT) prior to sequencing. The re-amplified PCR products were sequenced in both the forward and reverse directions (ABI BigDye Terminator chemistry, Australian Genome Research Facility). All chromatograms were analyzed using Codoncode Aligner, with sequences being aligned with MUSCLE and blasted on GenBank (http://www.ncbi.nlm.nih.gov/BLAST). Phylogenetic analyses of sequences were performed using maximum parsimony and maximum likelihood in MEGA 4 [59] under the delayed transition setting and calculation of bootstrap support values based on 1000 replicates.

### Host *atp6* sequence analyses

The mitochondrial atp6 gene (and parts of the flanking nad6 and nad4) was amplified for a subset of samples (n = 93: ~5 samples were randomly picked per species per depth) using the newly designed primers Aga-atp6-F1: GGCTTTATTTGGGGCTGAA and Aga-atp6-R1: CCCACAAAACCAAAGCACTTA. Primers were designed from the mitogenomes of *A*. *humilis* (NC008160) and *Pavona clavus* (NC008160) [60]. PCR amplifications were performed with 1.0 μl of DNA, 2.0 μl 10× PCR buffer (Invitrogen), 1.0 μl 50 mM MgCl_2_, 0.7 μl 10 mM dNTPs, 0.7 μl 10 mM Aga-atp6-F1, 0.7 μl 10 mM Aga-atp6-R1, 0.15 μl of Platinum Taq DNA Polymerase (Invitrogen) and dH_2_O water to a total volume of 20 ml per reaction. The cycling protocol was: 1 × 95°C (5 min); 30 × [95°C (30 s), 58°C (60 s), 70°C (90 s)]; 1 × 70°C (10 min). PCR products were purified, sequenced, aligned and analysed as specified for the *Symbiodinium* ITS2 sequences. Additional sequences were retrieved from GenBank to compare the atp6-based phylogenies with those based on the mitochondrial cytochrome oxidase 1 (COI) and the nuclear 28S region [60-63].

Phylogenetic analyses of sequences were constructed using maximum likelihood (ML), maximum parsimony (MP) and Bayesian (BAY) methods through the programs MEGA5 (Tamura et al. 2011) and MrBayes (Huelsenbeck and Ronquist 2001) respectively. The best-fit model of molecular evolution was selected by hierarchical Akaike information criterion (AIC) using jModeltest [64] with a GTR model with invariant sites best describing the atp6 data under a log likelihood optimality criterion. For the COI and 28S sequences retrieved from GenBank, a GTR model with gamma-shaped variation and invariant sites best described the data of both these regions. Maximum likelihood analyses were performed using 1000 non-parametric bootstrap replicates. Bayesian analyses were performed with the Markov Chain Monte Carlo search run with 4 chains for 10^6^ generations, with a sample frequency of 100 generations and a “burn-in” of 2500 trees.

### Statistics

The contributions of taxonomic species, depth and *Symbiodinium* type on host genotypic variability (*atp6*) were assessed for positively identified *Agaricia* specimens under the AMOVA framework using GenALEx V5 [65], with either depth or *Symbiodinium* type nested within taxonomic species. In host species for which multiple *Symbiodinium* profiles were observed, an (nested) analysis of similarity (ANOSIM) was carried out using Bray-Curtis Distance in the software package Primer v6 to test for differences between sampling years (Two-way ANOSIM with depth nested within year) and depths (One-Way ANOSIM comparing individual depth groups).

## Results

### Species abundances over depth

The species abundances at Buoy Zero/One differed among depths and revealed different distribution ranges for the five agariciid species (Figure 1c). At the shallowest depths (2-5 m), *A*. *humilis* is the most dominant species, with *A*. *agaricites* starting to occur at 5 m. At 10-15 m, *A*. *agaricites* becomes the most dominant species, with colonies of *A*. *humilis* and *A*. *lamarcki* being present in lower abundances. At 25 m depth *A*. *agaricites* and *A*. *lamarcki* occur in roughly equal abundances, but *A*. *lamarcki* takes over as the dominant species beyond this depth. From 40 m onwards, *A*. *grahamae* starts occurring, although in low numbers. *H*. *cucullata* was observed from 10-60 m, but always in relatively lower abundances.

### Symbiont diversity across host species and depth

**Figure 2:**
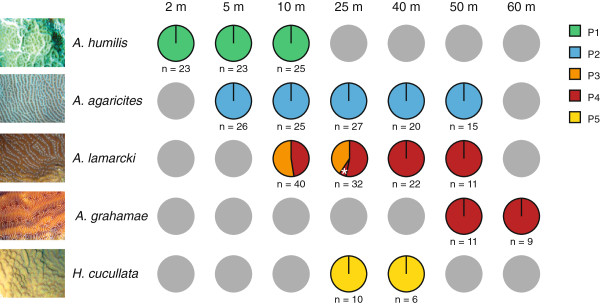
**Distribution of *Symbiodinium *ITS2 profiles across the five agariciid host species and depths.** Each pie chart represents the sampled population of a host species at a certain depth. Asterisk (*) indicates a P4 profile that had an additional C1 band. Figure legend text.

Across the five different host species, a total of six distinct ITS2-DGGE profiles were distinguished (Figure 2; see Additional file 1 for DGGE profiles). A distinct profile was observed for *A*. *humilis* (P1, n = 71), *A*. *agaricites* (P2, n = 113), and *H*. *cucullata* (P5, n = 16). Three different profiles (P3, n = 34; P4, n = 69; P4*, n = 2) were observed for *A*. *lamarcki*, of which one (P4) was shared with the species *A*. *grahamae* (n = 20) (Figure 2). Given that five of the six profiles contained at least three co-dominant ITS2 sequences, we decided to assign a number to the DGGE profiles (P1, P2, P3, P4, P4* and P5) rather than referring to individual *Symbiodinium* types. Although these co-dominant sequences likely represent intra-genomic variants within the rDNA of a single symbiont lineage (little variation in relative band intensity was observed) [66], it cannot be excluded that some of the profiles may represent a mix of distinct *Symbiodinium* types. Additionally, other *Symbiodium* types may be present at background levels that cannot be detected due to the limitations of ITS2-DGGE [57,58].

**Figure 3:**
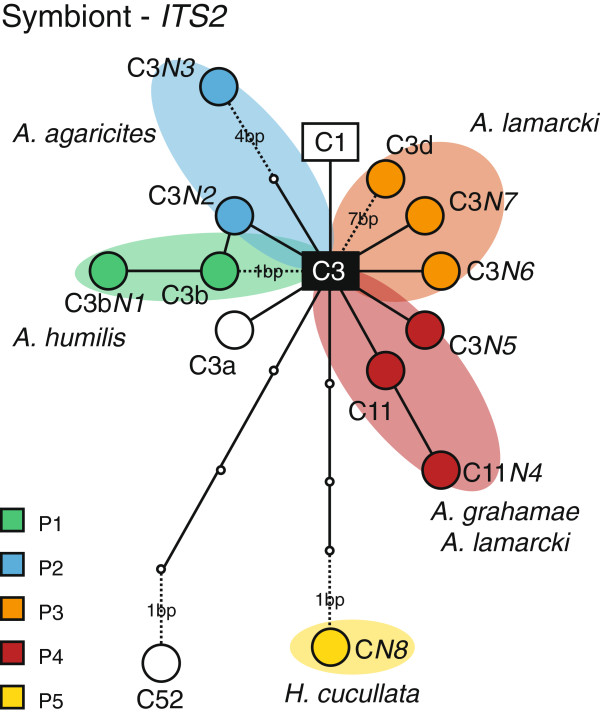
**Sequence network of *Symbiodinium *ITS2 types observed for each DGGE profile obtained from agariciid species.** Ancestral *Symbiodinium* types C1 and C3 are represented by squares. Circle size is not representative of frequency. Dashed lines indicate sequence gaps (with gap size in number of base pairs). Colours group the different ITS2 sequences observed in each DGGE profile. The C3 type (indicated by black square) is found in all DGGE profiles except for P5.

All the ITS2-DGGE fingerprint profiles for *Agaricia* spp. contained the *Symbiodinium* C3 sequence, in combination with 2-3 sequences that were all closely related to C3 (Figure 3). All of these sequences were novel (GenBank Accession Numbers KF551185-KF551192), except for C3b in *A*. *humilis*, C3d in *A*. *lamarcki*, and C11 in *A*. *lamarcki*/*grahamae*. Novel sequences are identified (in this manuscript) by a nomenclature that specifies the sequence to which they are most related, followed by a capital N (indicating novel sequence) and an arbitrary number in italics (e.g. C3b*N1*). Except for the shared C3 sequence, all profiles contained distinct *Symbiodinium* sequences (i.e. no overlap between profiles) indicating that each of these profiles contain (a) different *Symbiodinium* type(s). However, one *Symbiodinium* ITS2 profile (P4*) was observed in only 2 colonies of *A*. *lamarcki* (at 25 m) and was identical to the P4 profile but with an extra band that corresponded with the C1 sequence. The several *Agaricia* morphs that could not be unambiguously identified (*Agaricia* sp.) all contained the P4 profile (n = 10). For *H*. *cucullata* we could only obtain a single recoverable ITS2 sequence (C*N8*), which represented a more distant, novel *Symbiodinium* type (GenBank Accession Number KF551192) (Figure 3).

*A*. *humilis*, *A*. *agaricites*, *A*. *grahamae* and *H*. *cucullata* all harboured a single symbiont profile across the entire depth range across which they were sampled. *A*. *lamarcki*, the only species observed to harbour multiple symbiont profiles, showed a marked zonation, with colonies at 10 and 25 m depth harbouring either P3 (n = 34) or P4 (n = 36), whereas colonies (>40 m) exclusively harboured P4 (n = 33). Colonies hosting a mix of P3 and P4 profiles were not observed. There were no significant differences (Two-Way ANOSIM; depth nested within sampling year) between sampling years. The pairwise comparisons between the different depths confirmed that the symbiont community associated with *A*. *lamarcki* at 40 m was significantly different from 10 and 25 m (One-Way ANOSIM; not taking into account sampling year; respectively R = 0.15; p < 0.01 and R = 0.082; p < 0.05).

### Host genetic structure across taxonomic species and depth

**Figure 4:**
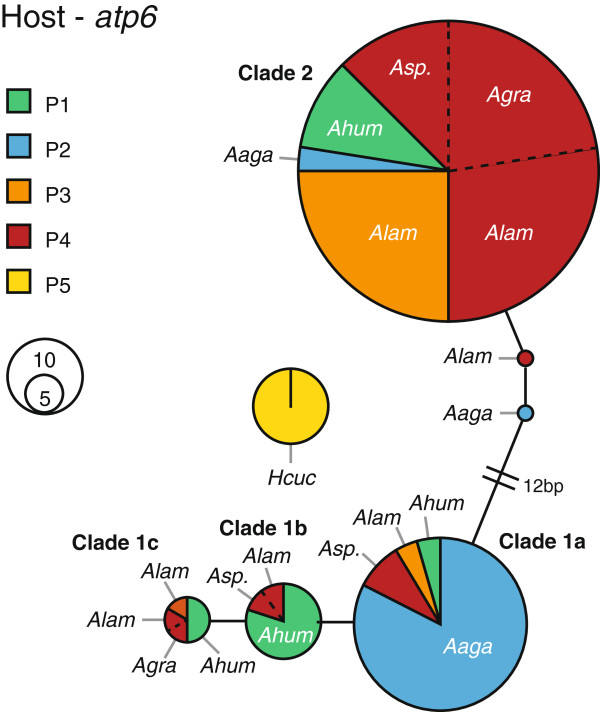
**Unrooted network of host *atp6 *mitochondrial haplotypes.** Circle size indicates the relative frequency of each haplotype. Pie chart indicates for which agariciid species the haplotype was observed (Ahum = *A*. *humilis* (n = 16), Aaga = *A*. *agaricites* (n = 22), Alam = *A*. *lamarcki* (n = 27), Agra = *A*. *grahamaei* (n = 9), Asp. = Unidentified *Agaricia* species (n = 9), Hcuc = *H*. *cucullata* (n = 10)), and colour indicates the *Symbiodinium* profile that was hosted.

Seven different mtDNA haplotypes were identified across the 93 specimens for which the *atp6* region (1260 bp including indels) was sequenced (Figure 4; GenBank Accession Numbers KF551193-KF551199). A single haplotype was observed for *H*. *cucullata* (n = 10), and indels were only observed when *Agaricia* sequences were aligned with those of *H*. *cucullata*. The remaining haplotypes were observed for *Agaricia* specimens and the number of substitutions between these sequences ranged from 0 to 16. The *Agaricia* haplotype network consists of two clades and four main haplotypes (i.e. subclades), with the majority of *A*. *lamarcki* and all *A*. *grahamae* specimens belonging to clade 2 (respectively 90 and 100%), most *A*. *agaricites* belonging to subclade 1a (86%), and *A*. *humilis* belonging to subclades 1b and 1c (69%). The *Agaricia* sp. specimens that were sequenced (n = 9 of 10) were found across the two main clades. Under the AMOVA framework (including only identified *Agaricia* specimens), 60% of molecular variance (Φ_SPP-TOT_ = 0.599; P < 0.001) was explained by morphotaxonomic species designation. No significant contributions of *Symbiodinium* type (2%; Φ_SYM-SPP_ = 0.058; P = 0.232) and depth (0%; Φ_DEP-SPP_ = -0.059; P = 0.668) could be detected, when nested within taxonomic species.

**Figure 5:**
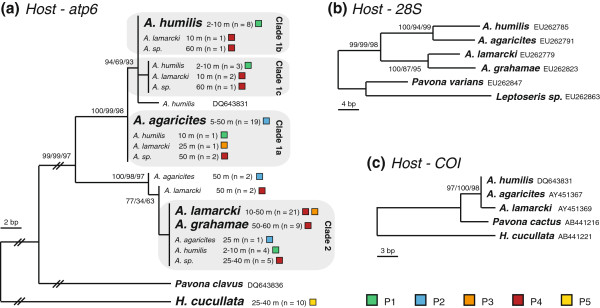
**Phylogenetic trees of *Agaricia *spp. based on mitochondrial and nuclear markers.** Phylogenetic trees (maximum parsimony) of *Agaricia* spp. based on **(a)** mitochondrial *atp6* (this study), and **(b)** nuclear 28S and **(c)** mitochondrial COI (previous studies: Medina et al. 2006; Fukami et al. 2008; Shearer and Coffroth 2008; Barbeitos et al 2010). *Pavona* spp., *Helioseris cucullata* and *Leptoseris* sp. are used as outgroups. Bootstrap values are based on Bayesian (BAY), maximum parsimony (MP) and maximum likelihood (ML) respectively, with only probabilities over 50% shown. Depth range and number of specimens are mentioned for each species, coloured boxes indicates the *Symbiodinium* profile that was hosted. Sequences retrieved from GenBank are indicated by their Accession Number.

Phylogenetic analyses (ML, MP and BAY) of the *atp6* region of the *Agaricia* specimens (Figure 5), using *H*. *cucullata* and *Pavona clavus* as outgroups, supported the monophyly of the genus *Agaricia* and subdivision of the genus into two main lineages (with bootstrap values across phylogenetic methods higher than 97), with sequences of the two lineages largely corresponding with *A*. *lamarcki* / *A*. *grahamae* and *A*. *humilis* / *A*. *agaricites* specimens respectively. The latter lineage is further subdivided into two lineages largely corresponding with *A*. *humilis* and *A*. *agaricites* (ML/MP/BAY: 94/69/93). Despite the mostly invariant host sequences among conspecifics, a small number of specimens belonging to different species were observed within each lineage. However, in all these cases the symbiont type hosted by these specimens confirmed the taxonomic identity (see Figure 4). Specimens that could not be identified (*Agaricia* sp.) were found to belong to the three different groups, although all hosting *Symbiodinium* profile P4, with 5 specimens from 25-40 m in the *A*. *lamarcki*/*grahamae* group, 2 specimens from 50 m in the *A*. *agaricites* group, and 2 specimens from 60 m in the *A*. *humilis* group.

Analyses of COI and 28S sequences for the same *Agaricia* spp. [60-63], using *Pavona* spp. and respectively *Leptoseris* sp. and *H*. *cucullata* as outgroups, corroborate the phylogenetic pattern revealed through the *atp6* region (Figure 5b, c). However, where COI shows no differentiation between *A*. *humilis* and *A*. *agaricites*, the 28S region differentiates between *A*. *lamarcki* and *A*. *grahamae* specimens. As such, the 28S region supports the subdivision of the *Agaricia* genus into two main lineages, corresponding to shallow (*A*. *humilis* and *A*. *agaricites*) and deeper species (*A*. *lamarcki* and *A*. *grahamae*).

Despite this cladal division into shallow and deep *Agaricia* species, no clear genetic subdivision was observed within species over depth (Figure 5), or in the case of *A*. *lamarcki* in relation to symbiont type (Figure 4). Nonetheless, several shallow specimens of *A*. *lamarcki* were found within the *A*. *humilis* (n = 3; 10 m) and *A*. *agaricites* groups (n = 1; 25 m). Additionally, several specimens of *A*. *agaricites* from 25 (n = 1) and 50 m (n = 2) depth were observed in the *A*. *lamarcki*/*grahamae* lineage.

## Discussion

This study assessed the depth distribution and genetic variation of five agariciid coral species and their associated *Symbiodinium* across a large depth gradient (2-60 m) on a single reef. Overall, we found a tight coupling between coral species and their dominant *Symbiodinium* at the study location, and strong evidence for depth-related niche partitioning of these associations. Depth-related parameters appear to have played a major role in the diversification of the coral genus *Agaricia* and its dominant algal endosymbionts, resulting in a clear genetic partitioning between coral host-symbiont communities across shallow and mesophotic depths.

### *Symbiodinium* diversity and host-symbiont specificity

Associations between the coral host and its dominant algal endosymbionts (*Symbiodinium*) at the study location were found to be specific in that each of the five agariciid species harboured a distinct *Symbiodinium* profile (except for *A*. *lamarcki* and *A*. *grahamae* that had one profile in common) (Figure 2). The four *Symbiodinium* profiles found in association with the four *Agaricia* species all shared the C3 sequence as an intragenomic variant and contained closely related *Symbiodinium* sequences (Figure 3). This corroborates observations in other parts of the Caribbean (Bahamas, Barbados, Belize, Florida, Mexico, US Virgin Islands), where *Agaricia* species have been found in association with C3-related *Symbiodinium* types [16,46-50].

Nonetheless, except for the C3b-related *Symbiodinium* type in *A*. *humilis* (profile P1), the agariciids studied here hosted distinct *Symbiodinium* types compared to those previously reported (e.g. B1, C3, C3a, C3q, D1a) [16,46-50]. *A*. *lamarcki* associated with two profiles (P3 and P4) containing respectively a C3d and C11 sequence, which indicate *Symbiodinium* types related to those found in respectively *Montastraea* (C3d) and mussid and faviid genera such as *Scolymia*, *Mussa* and *Mycetophyllia* (C11) [16,35,46]. *H*. *cucullata* hosted a *Symbiodinium* type (C*N8*/profile P5) that is very distinct from the C3 type previously reported in association with this species in other parts of the Caribbean [46,48,50], and does not contain the C3 sequence as an intragenomic variant. Some of these differences in *Symbiodinium* associated with *Agaricia* spp. (other studies) can be contributed to geographic separation of populations from the Western and Eastern Caribbean [50], however it also demonstrates that differences in symbiont associations exist even at smaller geographic scales, such as within the Eastern Caribbean (i.e., between Curaçao and Barbados). These observations corroborate the notion that the level of host-symbiont specificity can vary across geographic locations [22,29,32], and therefore has to be assessed locally in terms of the ecological implications.

The maternal transmission of endosymbionts leads to relative isolation of host-specific *Symbiodinium* populations, and is likely to be responsible for the local host specificity and the geographic variation in host-symbiont associations, as *Symbiodinium* types coevolve locally with their coral partners [22]. Similar host-symbiont specificity has been observed in other coral genera with a vertical symbiont acquisition mode, such as in various pocilliporid genera (e.g. *Seriatopora*, *Pocillopora*, *Stylophora*, *Madracis*) and the genera *Montipora* and *Porites*[20,22-25,29]. Although the incongruence between host and symbiont phylogenies points towards the importance of occasional host-symbiont recombination [13,16,28,29], such recombination through “symbiont replacement” is unlikely to occur over ecological timescales [67]. Therefore, despite the variation in host-symbiont associations observed for Agariciids across the broader Caribbean, it remains questionable whether the agariciid species at the study location can respond to rapid environmental change due to the lack of standing variation (i.e. diversity) in *Symbiodinium* associations.

The host-symbiont specificity observed here relates to the dominant *Symbiodium* types that are hosted by each species, and high-resolution approaches to the detection of *Symbiodinium* (e.g. using quantitative PCR or next-generation sequencing) may demonstrate that these agariciid species also host non-specific background *Symbiodinium*[57,58]. In addition, we observed the generalist *Symbiodinium* type C1 in 2 out of 105 sampled specimens of *A*. *lamarcki* (in addition to profile P4), indicating that non-specific associations occasionally do occur [29]. Although the potential of “symbiont shuffling” (i.e. shift in dominant *Symbiodinium* within a coral colony over its lifespan) as a response to environmental change remains debated [67], unusual associations (such as the C1 hosted in *A*. *lamarcki*) may become more abundant if these exhibit higher levels of holobiont fitness under the predicted changing environmental conditions.

### Depth-partitioning of agariciid host species and associated *Symbiodinium*

The five agariciid coral species exhibited a clear partitioning across the depth-mediated environmental gradient, progressing from respectively *A*. *humilis*, *A*. *agaricites* and *A*. *lamarcki* as most abundant species from shallow to deep (Figure 1c). *A*. *humilis* is a clear “shallow-water specialist” largely restricted to the shallow reef terrace (2-10 m), whereas *A*. *grahamae* is a “deep-water specialist” that exclusively occurs in the mesophotic zone (40-60 m). In contrast, both *A*. *agaricites* and *A*. *lamarcki* are more generalistic in terms of their depth distribution, albeit that *A*. *agaricites* predominates at shallower depths (5-25 m) and *A*. *lamarcki* at greater depths (25-60 m), with 25 m representing a transitory depth between both species (Figure 1c). These depth-distributions are from a single study location, and may vary across locations with different environmental conditions (e.g. water clarity) [68]. However, similar zonation of *Agaricia* species has been reported across the Caribbean [37,38,69-72], with *A*. *humilis* and *A*. *agaricites* most abundant on the shallow end of the depth spectrum, and *A*. *lamarcki* and *A*. *grahamae* predominating at greater depths. *H*. *cucullata* was found across a large depth range (10-60 m), however only in very low abundances, reflecting the remarkable decline of this species on the reefs in Curaçao compared to ~30 years ago [44,45].

Four of the agariciid species hosted a single *Symbiodinium* type over their entire depth range. In contrast, the depth-generalist species *A*. *lamarcki* exhibited symbiont depth zonation, changing from hosting P3 (i.e. C3/C3d -related) or P4 (i.e. C3/C11-related) in the shallow to exclusively P4 at mesophotic depths. The “deep-water specialist” *A*. *grahamae* harboured this same P4 symbiont profile, indicating that this *Symbiodinium* type may be particularly well-adapted to deeper water conditions. Similar putative “deep-specialist” *Symbiodinium* types have been identified in the genera *Madracis* and *Montastraea*[21,34,35]. Conversely, the ability of *A*. *lamarcki* to associate with another *Symbiodinium* lineage (represented by the P3 profile) may facilitate its broader depth distribution (i.e., extending into the shallow) compared to *A*. *grahamae* (although the P4 profile was found at shallow depths in *A*. *lamarcki* as well). Symbiont zonation appears to be a common feature of depth-generalist coral species in the Caribbean [31], however the occurrence of a single *Symbiodinium* profile in *A*. *agaricites* from 5-50 m depth indicates that such zonation, however, is not a prerequisite for broad distribution patterns [27,73].

**Figure 6:**
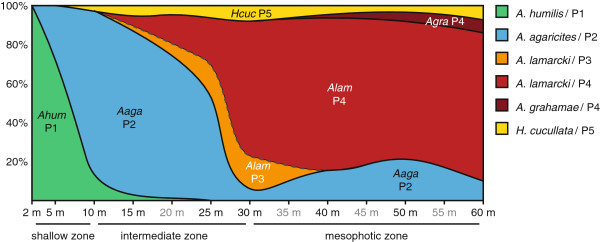
**Conceptual diagram depicting niche partitioning of agariciid host-symbiont assemblages over depth.** Relative abundances are extrapolated from average densities of host species and relative symbiont abundances across depths.

**Figure 7:**
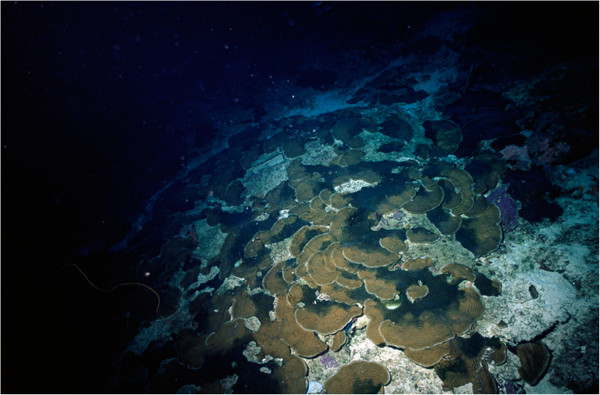
**Photo of *Agaricia *community beyond the depth range of this study.** Paper-thin *Agaricia grahamae* communities at 86 m depth on the leeward shore of Curaçao.

When considering both the average densities of host species and dominant symbiont associations across depths, it is obvious that host-*Symbiodinium* assemblages exhibit a clear niche partitioning over depth, for all assemblages apart from *H*. *cucullata* / P5 (conceptual diagram shown in Figure 6). Although both *A*. *humilis*/P1 and *A*. *agaricites*/P2 predominate in the shallow reef environment, *A*. *humilis*/P1 thrives in the shallowest of depths (2-10 m) whereas *A*. *agaricites*/P2 is most abundant at intermediate depths (10-25 m). As such, *A*. *humilis*/P1 is restricted to depths with high-irradiance conditions (87-50% of surface irradiance) and *A*. *agaricites*/P2 occurs in all but the brightest light conditions (1.5-70%). At greater depths (≥ 25 m), however, *A*. *lamarcki*/P4 becomes the most dominant host-symbiont assemblage. Given that all sampled colonies of *A*. *lamarcki* at 10 m depth (n = 40) were growing in cryptic locations on the reef (Bongaerts and Frade pers. obs.), the shallow end of *A*. *lamarcki*’s depth distribution is clearly determined by a maximum tolerable irradiance. Additionally, the *A*. *lamarcki*/P3 host-symbiont association, which only occurs at 25 m and cryptically at 10 m, occupies a niche that encompasses a very narrow light range. Instead, *A*. *grahamae* that shares symbiont profile P4 (C3/C11-related) with *A*. *lamarcki*, starts to appear at slightly greater depths than the *A*. *lamarcki*/P4 combination, indicating the potential (acclimatization) importance of the host component in mediating the distribution of this host-symbiont partnership [34]. Although *A*. *lamarcki* was found to be most abundant at the deeper end of the sampled depth spectrum (60 m), anecdotal observations at greater depths (65-85 m) indicate that *A*. *grahamae* may in fact become the more dominant species (Figure 7; Vermeij and Bongaerts, pers. obs.).

Although incident irradiance is likely an important factor to explain the observed depth partitioning of host-symbiont associations, other environmental variables, such as light spectral composition [74], temperature [75] and nutrient/plankton availability [26] may be important as well. For example, the depth range between 25 and 40 m appears to be a transition zone of strong genetic change, which corresponds to the depth range where influxes of deep oceanic water become noticeable in the temperature regime [21,39]. Given the various abiotic factors that vary over depth it remains impossible to evaluate the individual contributions of these factors in the observed partitioning, however light and temperature are likely to play important roles given their dramatic changes over depth at the study site [21,76]. While representing a massive undertaking, repeating similar symbiont genotyping efforts across a range of different locations would provide us with further insight into the role of different environmental conditions in the depth partitioning of host-symbiont associations.

### Host and symbiont (co) evolution

The mtDNA-based phylogenetic analysis showed that none of the *Agaricia* species studied is monophyletic, however, the presently accepted species designation can explain the majority of the observed molecular variance (~60%). Two major lineages were observed for the *Agaricia* specimens based on the *atp6* region, basically dividing the genus into a shallow (*A*. *humilis*/*A*. *agaricites* – Clade 1) and a deep (*A*. *lamarcki*/*A*. *grahamae* – Clade 2) species group. The *atp6* phylogeny is consistent with the phylogeny of the ribosomal 28S region (outgroup *H*. *cucullata* not included) produced with GenBank sequences, although the 28S region appears more variable (Figure 5). Although the *atp6* region does not separate *A*. *lamarcki* and *A*. *grahamae*, the phylogeny of the 28S region demonstrates that these are unlikely to represent synonyms. The *H*. *cucullata* clade is genetically very distinct and even shows (across markers) a closer relation to the Indo-Pacific genus *Pavona* than the Atlantic *Agaricia* genus (Figure 5). The phylogeny of *Agaricia*, forming two major clusters representing shallow and deep species, indicates that depth-related factors have played a major role in the original divergence within this genus. Given that also within each of these clusters, each species again exhibit distinct depth distributions on respectively the shallow and deep reef (Figure 1c), highlights the potential role of depth-related factors in the diversification within this genus throughout its evolutionary history.

Within the genus *Agaricia* all of the four major host atp6 haplotypes are, in fact, shared by more than one *Agaricia* taxon. This shared genetic polymorphism and repetitive non-monophyly has been shown in the past for many other coral taxa [9,77-81]. Such incongruence between morphospecies and genetically delimited phylogenies have been explained by several authors as being the result of phenomena such as morphological convergence (homoplasy), phenotypic plasticity, recent speciation with incomplete lineage sorting, morphological stasis, and/or interspecific introgressive hybridization [80]. Although introgressive hybridisation has been suggested in other brooding coral species [9,80,82], the fact that we only used a single mitochondrial marker prevents inference of such processes in the current study. The “misplaced specimens” in the phylogeny all hosted the *Symbiodinium* profile specific to that species (Figure 2), confirming that these are unlikely to represent taxonomic misidentifications. However, the observation that several shallower specimens of *A*. *lamarcki* (n = 4) grouped with the “shallow” species group (Clade 1a-c), and several deeper specimens of *A*. *agaricites* (n = 3) grouped with the “deep” species group (Clade 2) (Figure 5), could potentially be indicative of hybridization due to low densities of conspecific sperm.

The evolutionary divergence between “shallow” and “deep” *Agaricia* host species and genetic relatedness among them is reflected in the symbiont associations, with *A*. *lamarcki* and *A*. *grahamae* sharing an identical *Symbiodinium* profile (P4), and *A*. *humilis* and *A*. *agaricites* harbouring a related ITS2 sequence in their *Symbiodinium* profiles (C3b in P1 and C3*N*2 in P2) (Figure 3). This highlights, despite potential hybridisation within the *Agaricia* genus (which could result in the crossing over of *Symbiodinium* types), the diversification of both host and symbiont through reciprocal evolutionary changes (at least at a microevolutionary scale). These observations corroborate the growing body of evidence suggesting that corals with a vertical symbiont acquisition (i.e., usually brooding corals) and their associated, dominant *Symbiodinium* are involved in coevolutionary processes [8,9,26,29,83].

### A common pattern for Caribbean brooding corals?

The depth niche partitioning, host phylogenetic structure and local host-symbiont specificity observed for the genus *Agaricia* has strong similarities to that of the model genus *Madracis*, which has been extensively studied at the exact same study site [9,21,34,51,76,83-87]. The Caribbean genus *Madracis* comprises six common taxonomic species whose distributions are partitioned over the reef slope, including shallow (*M*. *mirabilis* and *M*. *decactis*) and deep (*M*. *carmabi* and *M*. *formosa*) specialists and two depth-generalist species (*M*. *pharensis* and *M*. *senaria*). However, similar to *A*. *lamarcki*, the distribution of *M*. *pharensis* and *M*. *senaria* is restricted to cryptic locations at depths of 10 m and shallower [51]. As such, the genus *Madracis* can be roughly divided into “shallow” species (*M*. *mirabilis* and *M*. *decactis*) and “deep” (*M*. *pharensis*, *M*. *senaria*, *M*. *carmabi* and *M*. *formosa*) species. This subdivision corresponds to the two main clades observed in the *Madracis* phylogeny based on the mitochondrial *nad5* region [9], similar to the genetic divergence observed between “shallow” and “deep” species in the genus *Agaricia*.

With regards to the associated *Symbiodinium*, the genus *Madracis* harbours three physiologically distinct but phylogenetically closely related symbiont types (B7, B13, B15), which are also partitioned over depth and to a certain extent specific to certain host species [21,34]. The shift in symbionts observed for *M*. *pharensis* corresponds to distinct physiological capacities of the symbionts involved [34], but also corresponds with a genetic divergence between shallow and deep populations of the host species *M*. *pharensis*[9]. Such intra-specific genetic divergence was not observed for *A*. *lamarcki* (the only *Agaricia* species that exhibited symbiont zonation), however it does corroborate the general pattern of strong host-symbiont coupling in *Agaricia* (this study).

Overall, the observed niche partitioning and evolutionary split between shallow and deep host lineages observed for the genera *Agaricia* and *Madracis*, highlight the role of the depth-related environmental gradient (i.e. differences in light, temperature, etc.) as a driving factor in the evolution of the congeneric species associated with these genera. The molecular phylogenies of both genera are relatively incongruent relative to the taxonomic classification based on morphological traits [9,84], highlighting the potential additional importance of introgressive hybridization. However, the role of introgressive hybridization in *Agaricia* is hard to infer given the use of a single, maternally-inherited marker region in this study. Nonetheless, the fact that species in both genera remain recognizable and have distinct ecological distributions does point towards the importance of divergent/disruptive selection across a depth gradient in the diversification of these genera. Given the prevalence of a brooding reproductive strategy [36] and vertical symbiont acquisition mode in the Caribbean, ecological adaptation to different ends of the depth spectrum may have played a major role in the diversification of many Caribbean corals.

### Implications for vertical connectivity

In recent years, there has been a growing interest in the role of deep reef areas as local refugia and a source of propagules [31], particularly given the observed relative stablity of some mesophotic reef environments [39] and accumulating evidence of localized recruitment on coral reefs [88-90]. The extent of genetic specialization within the genus *Agaricia* to both shallow and deep reef environments (Figure 6) indicates that, although the mesophotic *Agaricia* communities have remained relatively stable in the geographic location of our study [39], these communities are unlikely to provide significant amounts of propagules to ensure rapid shallow-reef recovery post-disturbance. The unrelated genus *Madracis*, shows a similar genetic segregation between its host-symbiont communities at mesophotic (dominated by *M*. *pharensis* / B15 and *M*. *formosa* / B15) and shallow depths [9,21], confirming that this pattern may extent across a broad range of coral genera. Nonetheless, the limited overlap between shallow and deep communities indicates that deep reef may still play an important role in ensuring local protection of certain genotypes (e.g. the small number of *A*. *agaricites* colonies that occur in the deep) when adjacent shallow-reefs get affected by a major disturbance.

## Conclusions

The findings in this study highlight that depth-related parameters have played a major role in the diversification of brooding corals and their associated *Symbiodinium*, and corroborate the notion that brooding corals and their *Symbiodinium* are engaged in processes of coevolution. Further studies should investigate whether any adaptive differentiation of these species has occurred at the population level, and whether the high levels of specificity observed here are also prevalent in other brooding coral genera. Overall, we need a better understanding of the actual mechanisms through which the depth-related environmental gradients have led to population/species divergence on coral reefs and how this evolutionary history affects the current day and future interconnectedness of shallow and deep reef systems.

## Competing interests

The authors declare that they have no competing interests.

## Authors’ contributions

PB designed and conceived of the study, collected specimens, carried out labwork, performed the genetic analyses, and drafted the manuscript. PRF designed and conceived of the study, collected specimens, carried out labwork, helped with the interpretation of data, and drafted the manuscript. JJO, KBH and NE collected specimens, carried out labwork and helped to draft the manuscript. JvB carried out labwork, and helped to draft the manuscript. MJHV carried out the transects, collected specimens and helped to draft the manuscript. RPMB, PMV, and OHG designed and conceived of the study and helped to draft the manuscript. All authors read and approved the final manuscript.

## Supplementary Material

Additional file 1 **Denaturing gradient gel electrophoresis of *Symbiodinium *ITS2 types associated with *Agaricia *species.** Sequences used to characterize each symbiont profile are shown adjacent to bands in the gel image. Types in italics represent novel sequences, with the name specifying the sequence to which they are most related, followed by a capital N (indicating novel sequence) and an arbitrary number (e.g. C3b*N1*). Click here for file
