# Supplementary figures and images for: Sharing the slope: depth partitioning of agariciid corals and associated Symbiodinium across shallow and mesophotic habitats (2-60 m) on a Caribbean reef
Source: BMC Evol Biol. 2013 Sep 23;13:205. doi: 10.1186/1471-2148-13-205 (PMC3849765; doi:10.1186/1471-2148-13-205)

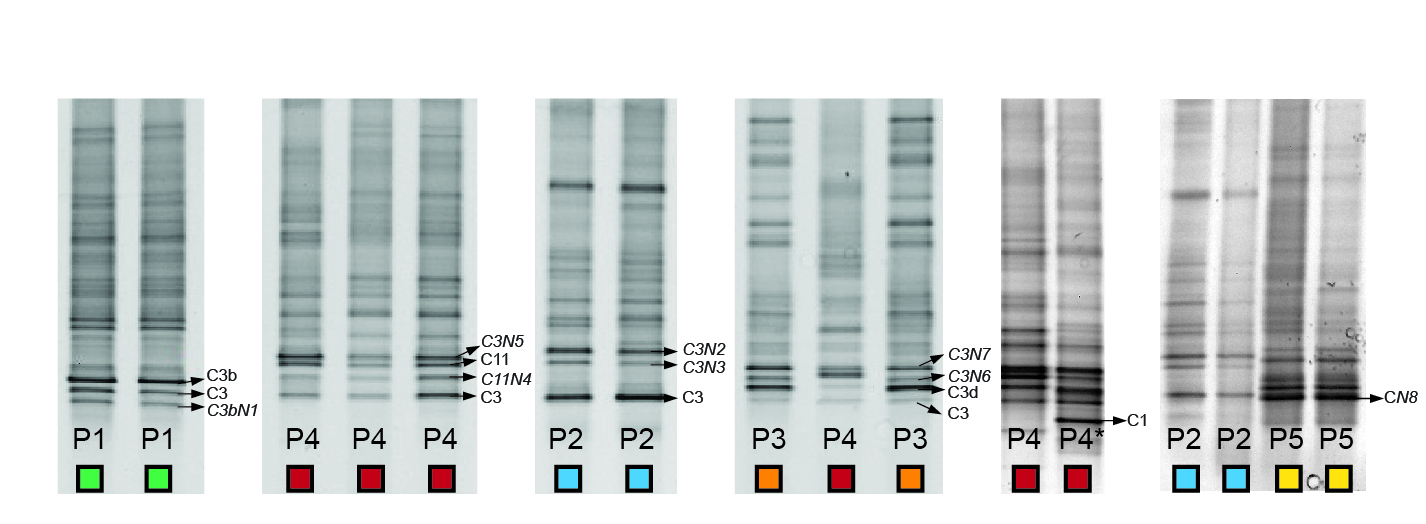

Supplement: Additional file 1 — Denaturing gradient gel electrophoresis of Symbiodinium ITS2 types associated with Agaricia species. Sequences used to characterize each symbiont profile are shown adjacent to bands in the gel image. Types in italics represent novel sequences, with the name specifying the sequence to which they are most related, followed by a capital N (indicating novel sequence) and an arbitrary number (e.g. C3bN1). [file 1471-2148-13-205-S1.jpeg]
